# Lower retention after retrograde coronary venous infusion compared with intracoronary infusion of mesenchymal stromal cells in the infarcted porcine myocardium

**DOI:** 10.1136/bmjos-2018-000006

**Published:** 2019-01-07

**Authors:** Wouter A Gathier, Mira van der Naald, Bas R van Klarenbosch, Anton E Tuinenburg, John LM Bemelmans, Klaus Neef, Joost PG Sluijter, Frebus J van Slochteren, Pieter A Doevendans, Steven AJ Chamuleau

**Affiliations:** 1 Department of Cardiology, Universitair Medisch Centrum Utrecht, Utrecht, Netherlands; 2 Department of Nuclear Medicine, Universitair Medisch Centrum Utrecht, Utrecht, Netherlands; 3 Regenerative Medicine Center Utrecht, Utrecht, Netherlands; 4 Department of Experimental Cardiology, Universitair Medisch Centrum Utrecht, Utrecht, Netherlands; 5 NL-HI (Dutch Heart Institute), Utrecht, Netherlands; 6 Central Military Hospital, Utrecht, Netherlands

**Keywords:** bmjos, mesenchymal stromal cells, myocardial infarction, stem cell transplantation

## Abstract

**Background:**

Commonly used strategies for cell delivery to the heart are intramyocardial injection and intracoronary (IC) infusion, both having their advantages and disadvantages. Therefore, alternative strategies, such as retrograde coronary venous infusion (RCVI), are explored. The aim of this confirmatory study was to compare cardiac cell retention between RCVI and IC infusion. As a secondary end point, the procedural safety of RCVI is assessed.

**Methods:**

Four weeks after myocardial infarction, 12 pigs were randomised to receive mesenchymal stromal cells, labelled with Indium-111, via RCVI (n=6) or IC infusion (n=6). Four hours after cell administration, nuclear imaging was performed to determine the number of cells retained in the heart both in vivo and ex vivo. Procedure-related safety measures were reported.

**Results:**

Cardiac cell retention is significantly lower after RCVI compared with IC infusion (in vivo: RCVI: median 2.89% vs IC: median 13.74%, p=0.002, ex vivo: RCVI: median 2.55% vs IC: median 39.40%, p=0.002). RCVI led to development of pericardial fluid and haematomas on the frontal wall of the heart in three cases. Coronary venous dissection after RCVI was seen in three pigs, of which one also developed pericardial fluid and a haematoma. IC infusion led to no flow in one pig.

**Conclusion:**

RCVI is significantly less efficient in delivering cells to the heart compared with IC infusion. RCVI led to more procedure-related safety issues than IC infusion, with multiple cases of venous dissection and development of haematomas and pericardial fluid collections.

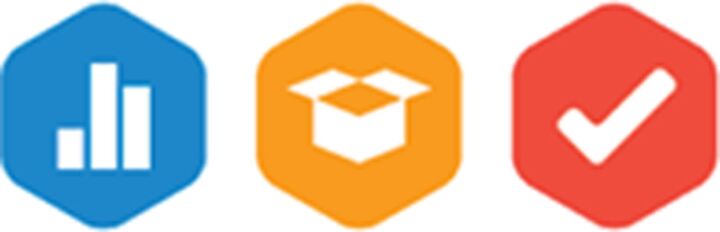

**Strengths and limitations of the study**

- To our knowledge, this is the first confirmatory study performed on cell retention after retrograde coronary venous infusion versus intracoronary infusion in a porcine model of chronic myocardial infarction.
- Adequate steps were taken to limit the risk of bias: the primary end point was prespecified; sample size was calculated beforehand to ensure adequate power of the study and prevent unnecessary use of animals.
- The study was performed in a randomised manner and outcome assessment was performed by blinded investigators.
- Radiolabelling with In^111^ made it possible to quantify cell retention in a very precise way.
- Precise determination of cell retention in the heart on total body images of pigs is challenging due to overprojection of the lungs and heart. This means counts coming from areas of the lungs that are positioned over the heart are attributed to the heart, leading to a slightly higher cell retention in the heart than was actually the case. Ex vivo measurements of cell retention were performed to overcome this drawback of in vivo imaging.

## Introduction

Cell therapy is suggested as a potential treatment option for ischaemic heart disease, yet only moderate improvement in cardiac function is achieved.^1 2^ The delivery of cells to the myocardium is an important limitation of current cell injection methodologies.^3^ The ideal strategy is safe, easy to perform and efficient in cell delivery. Intracoronary (IC) infusion and intramyocardial (IM) injection have been thoroughly tested.^4–7^ Both techniques present with disadvantages such as the need for patent coronary arteries and the risk of embolisation leading to decreased blood flow in case of IC infusion.^8–10^ The intramuscular injection procedure is time-consuming and requires specialised equipment in the catheterisation laboratory. Furthermore, rapid loss of cells via venous drainage is seen after IM injection.^11^ Alternative delivery strategies could possibly overcome these drawbacks. Retrograde coronary venous infusion (RCVI) is less commonly applied, but could be a good alternative to IC infusion and IM injection. However, the available data on technical and safety aspects of RCVI are insufficient and incomplete. At present, there are not enough arguments to proceed with this technique in the clinical arena because well-designed confirmatory studies on retention rates and safety data are required to prove its value.^12^

With RCVI, cells are retrogradely infused in the coronary venous system, which is typically free of atherosclerotic disease, and therefore could potentially improve delivery to the target area compared with IC infusion. An important limitation of cardiac cell therapy is the retention of cells in the heart after delivery. IM injection and IC infusion show comparable retention rates of 10%–15%.^4 13 14^ However, there are only limited data available on safety and the retention of cells in the heart after RCVI in large animal models and in the clinical setting. Currently, no direct comparison is available on cardiac cell retention after RCVI versus IC infusion in the setting of chronic myocardial ischaemia. In view of future clinical trials it is important to determine whether RCVI is a good alternative to IC infusion. Therefore, the aim of this confirmatory study is to compare the retention rates of radiolabelled mesenchymal stromal cells (MSCs) in the heart after RCVI and IC infusion and provide an estimate of safety of RCVI in a porcine model of chronic myocardial infarction (MI). We did not aim to provide data on cardiac repair because animals were terminated 4 hours after cell infusion to enable ex vivo scintigraphy of different organs.

## Methods

### Ethical statement

All animals received care in compliance with the ‘Guide for the Care and Use of Laboratory Animals’, published by the National Institutes of Health (National Institutes of Health publication 85–23, revised 1985). It was not possible to perform this experiment without animals due to the fact that the haemodynamics and biological nature of the heart and the whole body cannot be replicated in such a way that the results of this study would be translatable to the real situation. We minimised the number of animals used by performing a sample size calculation beforehand. Refinement was done by using proven techniques, performed by trained personnel. Furthermore, maximum effort was put into ensuring the best conditions for the animals in terms of housing, enrichment and analgesia.

### Study design

MI was induced in 16 female Dutch Topigs pigs (Van Beek SPF varkensfokkerij B.V., Lelystad, The Netherlands). Pigs were selected as the preferred animal for this experiment because of the resemblance of the pig and human heart in terms of anatomy and haemodynamics. Animals that survived 4 weeks after MI (n=12) were randomised (1:1) to receive MSCs labelled with Indium-111 (In^111^) via RCVI (n=6) or IC infusion (n=6). Randomisation was performed using a closed envelope system. Nuclear imaging was carried out 4 hours after MSC delivery, after which the anaesthetised animals were euthanised by potassium chloride overdose. Nuclear imaging data were analysed by lab technicians blinded to the infusion procedure.

The protocol of this study was registered on https://www.preclinicaltrials.eu/ (PCTE0000104) and the Animal Research: Reporting of In Vivo Experiments (ARRIVE) guidelines were followed for reporting. Heart rate, mean arterial pressure, left ventricular (LV) internal diameter at diastole and systole (LVIDd, LVIDs) were determined prior to MI (baseline) and directly prior to cell infusion.

### Experimental outcomes

**Figure 1:**
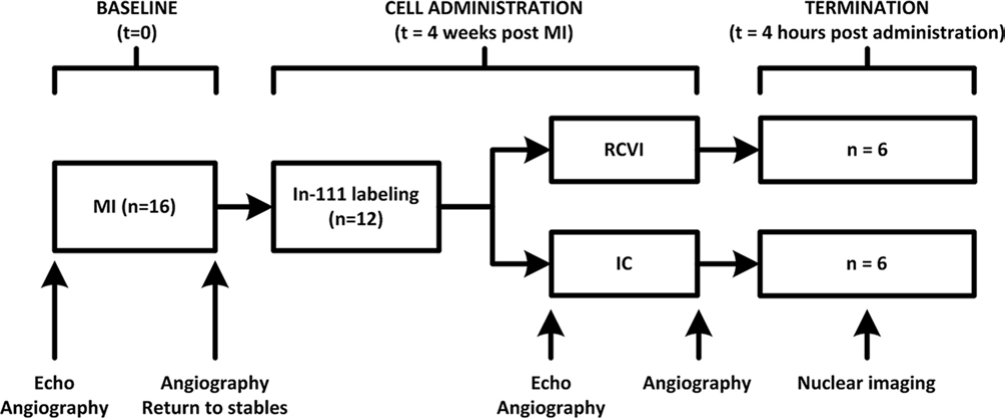
Experimental set-up. IC, intracoronary infusion; MI, myocardial infarction; n, number of animals; RCVI, retrograde coronary venous infusion; t, time point.

The primary end point of this study is retention of radiolabelled cells in the heart 4 hours after delivery. Cell retention was determined in vivo and ex vivo. In vivo analysis was performed by nuclear total body imaging of the live pig after which the percentage of total radioactive signal (counts) coming from the heart was divided by the total radioactive counts coming from the total body of the pig, including the bladder catheter. Because the heart is partially superimposed over the lungs during total body scanning, termination of the pigs and ex vivo scanning of individual organs (heart, lungs, kidneys, liver, spleen) was performed directly after in vivo scanning to check whether this superposition would influence the results of the total body imaging. The total radioactive signal (counts) coming from the heart was then divided by the sum of all radioactive counts coming from all aforementioned organs. The secondary end point is safety in terms of procedure-related complications such as occurrence of vessel dissections, flow obstruction during or after cell administration, development of pericardial effusion, and development of haematomas on the LV wall. Experimental set-up is shown in figure 1.

### Experimental procedures

#### Anaesthesia and analgesia

Prior to MI induction, all animals received a Butrans 5 µg/h patch. Animals were pretreated with amiodarone (1200 mg/day, 7 days), clopidogrel (75 mg/day, 3 days) and carbasalate calcium (loaded with 320 mg, 1 day), which was continued until the end of the experiment (daily dose 80 mg). Premedication (ketamine 10–15 mg/kg, midazolam 0.7 mg/kg and atropine 0.5 mg) was delivered intramuscularly. Anaesthesia was induced with thiopental sodium 4 mg/kg delivered through the ear vein. General anaesthesia and analgesia were maintained with a bolus of midazolam 10 mg and sufentanil 0.25 mg followed by intravenous delivery of midazolam 1 mg/kg/h, sufentanil 10 µg/kg/h and pancuronium bromide 0.1 mg/kg/h. Animals received 300 mg amiadarone in 500 mL venofundin 6% infused in 30 min. Mechanical ventilation was performed using a mixture of O_2_ and air (1:2) with a tidal volume of 10 mL/kg with 12 breaths per minute. Animals received 5000 IU of heparin every 2 hours during the procedure.

#### Myocardial infarction

MI was induced percutaneously by a temporal (90 min) occlusion of the left anterior descending artery (LAD) using an angioplasty balloon. The preferred occlusion site was after diagonal branch 2, but the infarct site was determined per pig based on the anatomy of the coronary arteries (thickness and tract). In case of ventricular fibrillation (VF) or ventricular tachycardia without output, 200-joule shocks were delivered using an external defibrillator in order to restore sinus rhythm. Chest compressions were given between shocks to maintain circulation. In addition, amiadarone (maximum of 3 times 150 mg), adrenalin (0.1 mg) and/or atropine (0.5 mg) were administered. Arterial blood pressure, ECG and capnogram were monitored during the entire procedure.

#### MSC culture and In^111^ labelling

Allogeneic MSCs were isolated and cultured in Minimal Essential Media with *alpha* modifications (αMEM) (Invitrogen, Carlsbad, California, USA) supplemented with 10% fetal bovine serum, 0.2 ng/mL vitamin C (Sigma-Aldrich, St. Louis, Missouri, USA), 1 ng/mL basic fibroblast growth factor (Sigma-Aldrich, St. Louis, Missouri, USA) and 1% penicillin/streptomycin. The cells were incubated at 37°C and medium was changed every 3 days. Cells were cultured in a 75 cm^2^ flask and passaged when they reached confluence, until passage 2–3. MSCs were frozen in 10% dimethylsulfoxide and 90% culture medium. Characterisation of MSCs was performed as previously described.^15 16^ Seven days prior to transplantation, MSCs were thawed, plated in flasks and grown to confluence, until passage 5–7. At the day of cell delivery, 10^7^ MSCs were labelled with carboxyfluorescein succinimidyl ester (CFSE) (Invitrogen, Carlsbad, California, USA) dissolved in dimethyl sulfoxide (DMSO) (Sigma-Aldrich, St. Louis, Missouri, USA) to a concentration of 5 mM after which cells were trypsinised and subsequently labelled with a median of 36.3 (IQR 33.5–40.5) megabecquerel (MBq) of In^111^ at 37°C for 20 min. After incubation, cells were washed up to three times with Hank’s balanced salt solution CaCl_2_+MgCl_2_+ (Life Technologies Corp, Grand Island, New York, USA) to remove unbound label. Radiolabel uptake efficiency was measured with a dose calibrator. After labelling, cell viability was assessed via trypan blue (Sigma-Aldrich, St. Louis, Missouri, USA) counting. Before injection, MSCs were resuspended in 10 mL phosphate buffered saline pH 7.4 (Life Technologies Corp, Grand Island, New York, USA).

The protocol on labelling of MSCs with In^111^ can be found at: https://www.protocols.io/view/labeling-of-porcine-mesenchymal-stromal-cells-mscs-mr9c596.

#### Histochemistry

Directly after termination, representative myocardial tissue samples were collected from areas of the heart that showed activity during nuclear imaging and were snap frozen in liquid nitrogen. Tissue samples were cut with the Cryostar NX70 (ThermoFisher, Waltham, Massachusetts, USA) at 10 µM. The EVOS FL (ThermoFisher, Waltham, Massachusetts, USA) cell imaging system was used to check for CFSE positivity. Histological samples were subsequently fixed with acetone, permeabilised with 0,1% Triton X-100 (Sigma-Aldrich, St. Louis, Missouri, USA) and blocked with 10% normal goat serum S-1000 (Vector Laboratories, Burlingame, California, USA). Monoclonal anti-α-actinin (sarcomeric) mouse antihuman (Sigma-Aldrich, St. Louis, Missouri, USA) (1:350) was used as the primary antibody followed by the secondary antibody goat antimouse-568 (1:350) (Invitrogen, Carlsbad, California, USA) and 1 µg/mL Hoechst 33 342 (Invitrogen, Carlsbad, California, USA). Samples were mounted with fluormount-G (ThermoFisher, Waltham, Massachusetts, USA). Imaging was performed with a confocal Leica SP8X microscope (Leica, Amsterdam, Netherlands).

#### Retrograde coronary venous infusion

Two different infusion catheters were used for RCVI. In case the coronary sinus (CS) was ≥5 mm in diameter a dedicated CS infusion catheter was used (Advance CS Infusion Catheter, Cook Medical, Bloomington, Indiana, USA). In case the diameter of the CS was <5 mm, an over-the-wire balloon catheter (Advance 35LP Low-Profile PTA Balloon Dilatation Catheter, Cook Medical, Bloomington, Indiana, USA) was used. Balloons were inflated at low pressure (maximum of 2 atmospheres) in the CS after which a venogram was made to ensure total occlusion of the CS. When total occlusion was observed, 2 mL of cell suspension followed by 8 mL of sodium chloride 0.9% was infused during 60 s. This procedure was performed a total of five times in order to infuse a total of 10 mL of cell suspension flushed with 40 mL of sodium chloride 0.9% in 5 min. Occlusion of the CS was maintained for 10 minutes after infusion to prevent washout of cells.

#### IC infusion

IC infusion was performed by placing an over-the-wire balloon (Emerge over-the-wire PTCA dilatation catheter, Boston Scientific Corp, Natick, Massachusetts, USA) of equivalent size to the LAD at the same site where occlusion was created during MI induction. After inflation of the balloon at low pressure, 3.3 mL of cell suspension was infused in 30–45 s. The balloon was deflated after 3 min to reinstate flow. After 3 min of flow, the procedure was repeated another two times to infuse a total of 10 mL of cell suspension.

#### Nuclear imaging and analysis

**Figure 2:**
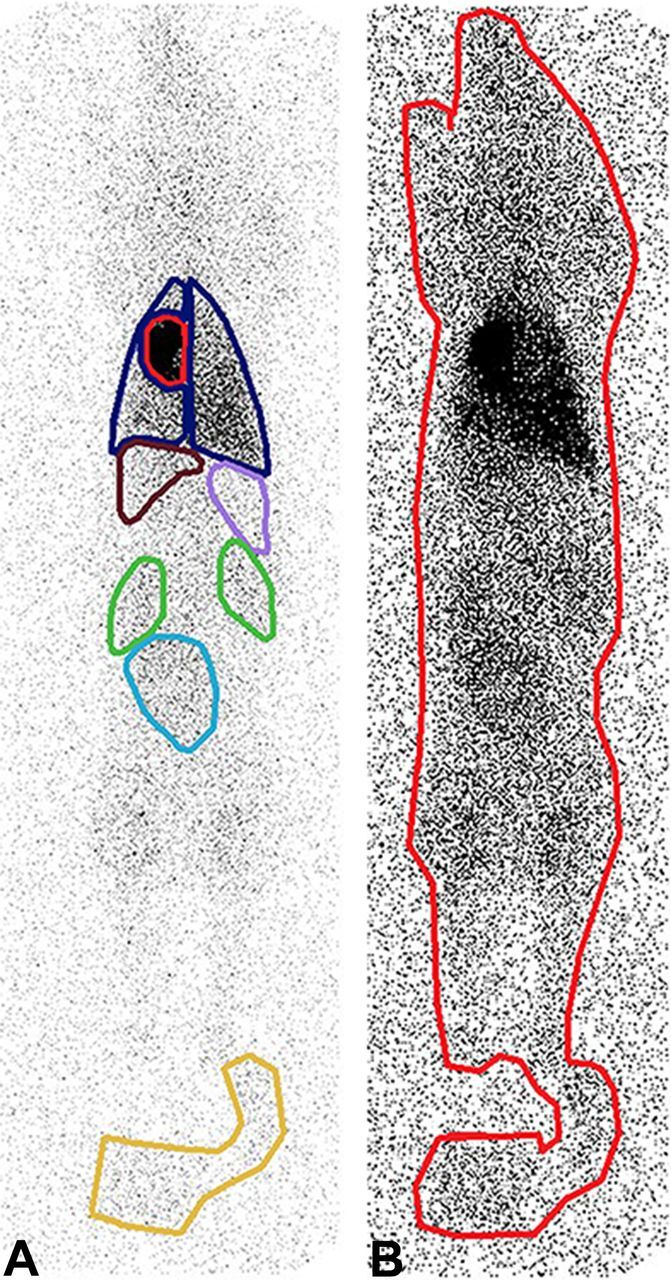
Total body scintigraphy with regions of interest (ROIs). (A) ROIs placed over visceral organs (heart in red, lungs in blue, kidneys in green, liver in brown, spleen in pink, bladder in light blue) and catheter bag in yellow. (B) ROI placed over total body of pig including catheter bag. Of note: both image A and image B are anterior captures. Both anterior and posterior images were captured for each animal and the number of counts used for analysis was based on the geometrical mean of the anterior and posterior counts.

In vivo and ex vivo scintigraphy was performed 4 hours after MSC administration using a dual head gamma camera (Phillips Skylight). A whole-body scan was acquired at both 174 keV and 247 keV energy windows using the following imaging parameters: medium-energy general-purpose collimator and 512×1024 projection matrix. The retained activity in syringes was measured with a dose calibrator (Azbil Telstar Benelux). Both anterior and posterior images were captured for each total body scan (in vivo) and each individual organ (ex vivo). The number of counts used for analysis was based on the geometrical mean of the anterior and posterior counts. After in vivo scanning, regions of interest (ROIs) were placed over the major visceral organs and total body of the pig (figure 2), using manufacturer’s software (JETStream workspace; Philips, Best, The Netherlands). The retention of In^111^-labelled cells in the heart was calculated as a ratio of the total radioactive signal (counts) coming from the heart divided by the total counts coming from the total body of the pig (including bladder catheter), after correction for anatomy. After ex vivo scanning of individual organs, the retention of In^111^-labelled cells in the heart was calculated as a ratio of the total radioactive signal (counts) coming from the heart divided by the total counts coming from all individual organs combined. Data analysis was performed by two to three laboratory analysts per animal coming from a pool of four analysts, supervised by an expert analyst, all blinded for treatment allocation.

#### Echocardiography

Transthoracic echocardiography (X5-1 probe, IE-33, Philips, Best, The Netherlands) was performed directly before MI induction and 4 weeks later, directly before MSC infusion. Chamber dimensions (LVIDd and LVIDs) were obtained in short-axis view at mid-papillary level. Analysis was performed in a blinded fashion by a trained physician.

### Experimental animals

#### Sample size

A total number of 12 animals (median age and weight at time of MI: 20 weeks (IQR: 18–22) and 72 kilograms (IQR: 68–76), respectively) was allocated to receive MSCs via either RCVI (n=6) or IC (n=6) infusion. This sample size was predefined, and calculated for an α of 0.05, power of 80%, maximum SD of 4% and an expected maximum absolute difference in cell retention of 7.5%. Because 4 animals died during or after MI induction, a total of 16 animals had to be used to include 12 animals in the analysis.

#### Housing

Animals were housed in stables with up to two pigs in the same stable before MI. After MI, animals were housed in separate stables to minimise stress. Animals were still able to see, smell and hear each other through the grates that divide the stables. Straw was used for bedding and environmental enrichment was provided in the form of special rods that the animals could nibble on and play with. Welfare was assessed daily by animal caretakers.

### Statistical analysis

Statistical analysis was performed using IBM SPSS statistics V.25 (IBM, Armonk, New York, USA). Baseline characteristics and cell retention are presented as median with IQRs. Comparison of data between two groups was performed using Mann-Whitney U test. A value of p <0.05 was considered statistically significant.

## Results

### Procedural data

VF during MI induction occurred in 13 out of 16 pigs, of which 2 died due to refractory VF. Another two pigs died in the stables due to acute heart failure or a heart rhythm disorder (day 4 and day 19) as a result of the MI. The remaining 12 pigs were randomised to RCVI (n=6) or IC infusion (n=6). No significant differences in heart rate, mean arterial pressure, LVIDd and LVIDs were seen between groups as seen in table 1A, although a trend was seen towards a larger LVIDs in pigs that were allocated to IC infusion both at baseline and at follow-up.

**Table 1:** (A) Heart rate (HR), mean arterial pressure (MAP), left ventricular internal diameter at diastole (LVIDd) and systole (LVIDs) before myocardial infarction (baseline) and directly prior to cell infusion. (B) Cell viability, total administered cells and total administered live cells. Values are depicted as median with IQR

1A Parameter	Baseline			Prior to cell infusion		
	IC (n=6)	RCVI (n=6)	P value	IC (n=6)	RCVI (n=6)	P value
HR (beats/min)	71 (63–81)	72 (66–73)	0.937	73 (64–82)	69 (63–76)	0.589
MAP (mm Hg)	74 (67–89)	70 (64–88)	0.699	79 (72–87)	75 (72–82)	0.699
LVIDd (mm)	49 (48–50)	47 (46–49)	0.342	58 (47 –59)	58 (47–62)	0.985
LVIDs (mm)	35 (34–37)	32 (29–34)	0.063	45 (40 –51)	38 (37–38)	0.115

**Table:** 

1B Parameter	IC (n=6)	RCVI (n=6)	P value			
Cell viability after labelling (%)	66.8 (62.1–72.4)	53.6 (49.8–73.8)	0.418			
Total administered cells (x 10^6^)	3.2 (3.2–3.7)	2.8 (2.1–3.1)	0.180			
Total administered live cells (x 10^6^)	2.4 (1.6–2.4)	1.6 (1.3–1.7)	0.167			

IC, intracoronary; RCVI, retrograde coronary venous infusion.

### Cell viability and numbers

The median viability of MSCs after labelling with In^111^ was 66.8% (IQR: 62.1–72.4) in the IC group versus 53.6% (IQR: 49.8–73.8) in the RCVI group (p=0.418). The median total administered cells was 3.2 M (IQR: 3.2–3.7) in the IC group versus 2.8 M (IQR: 2.1–3.1) in the RCVI group (p=0.180) The median number of administered live cells was 2.4 M (IQR: 1.6–2.4) in the IC group versus 1.6 M (IQR: 1.3–1.7) in the RCVI group (p=0.167). Results are shown in table 1B.

### Cell retention

#### In vivo analysis

A significant difference in MSC retention in the heart was seen between the RCVI and IC infusion groups with a median retention of 2.89% (IQR: 2.14–3.86) in the RCVI group versus 13.74% (IQR: 10.20–15.41) in the IC infusion group (p=0.002).

**Figure 3:**
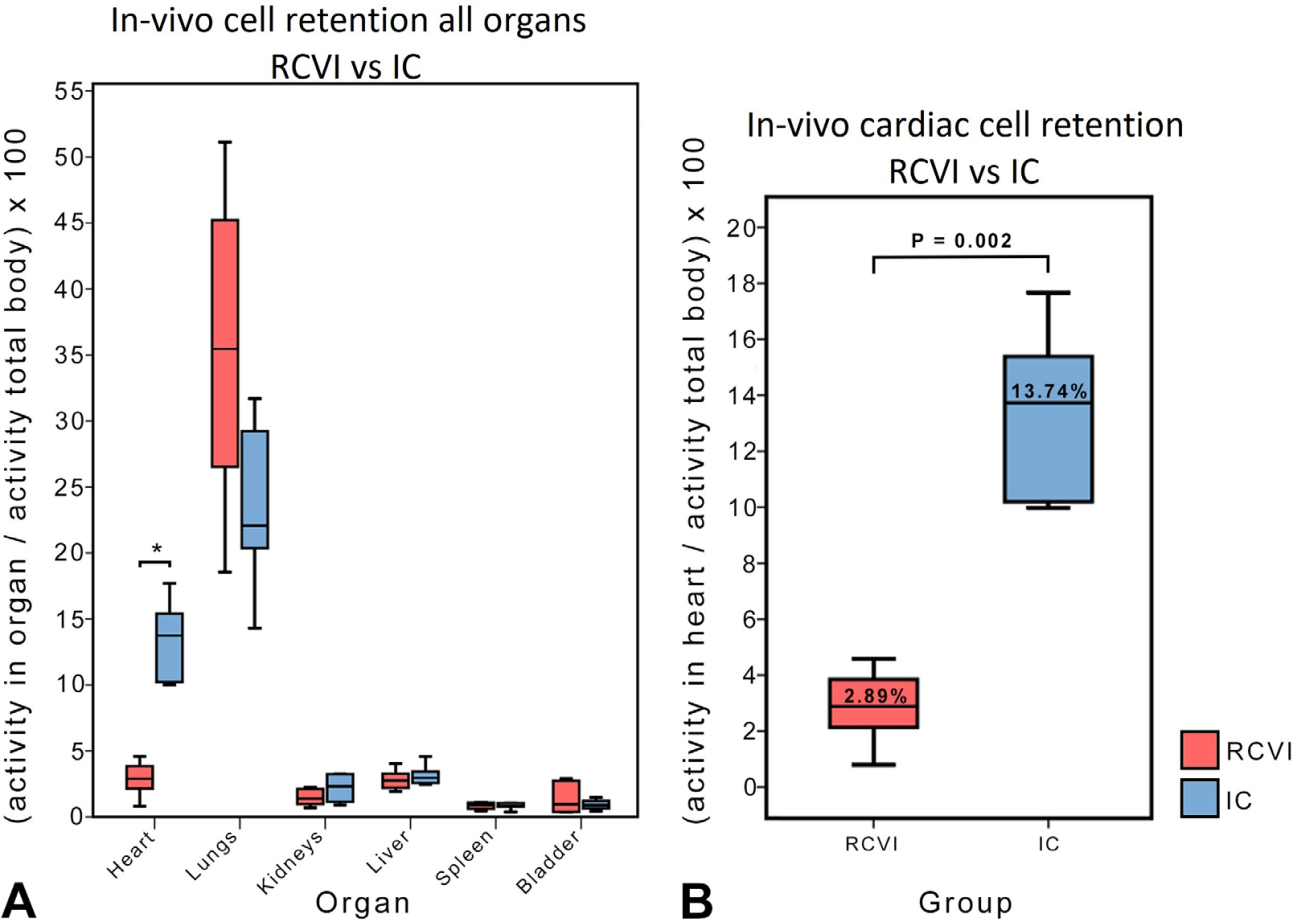
In vivo retention of cells in major organs presented as a percentage of total body activity. (A) In vivo analysis of activity in heart, lungs, kidneys, liver, spleen and bladder presented as a percentage of total body activity: retrograde coronary venous infusion (RCVI) versus intracoronary (IC) infusion. Only activity in the heart differed significantly between RCVI and IC infusion (*=p=0.002). (B) Magnification of figure 3A. Retention of mesenchymal stromal cells (MSCs) in the heart is significantly worse after RCVI compared with IC infusion.

No significant differences in cell retention were seen in lungs, kidneys, liver, spleen and bladder between RCVI and IC infusion, although a trend was seen towards higher retention of cells in the lungs after RCVI. Data are presented in table 2 and figure 3A, B.

**Table 2:** In vivo analysis of activity in heart, lungs, kidneys, liver, spleen and bladder as a percentage of total body activity. Values are depicted as median with IQRs

Organ	Median activity (%) (IQR)	Median activity (%) (IQR)	P value
	RCVI (n=6)	IC (n=6)	
Heart	2.89 (2.14–3.86)	13.74 (10.20–15.41)	0.002
Lungs	35.45 (26.53–45.22)	22.07 (20.36–29.22)	0.132
Kidneys	1.39 (0.97–2.12)	2.32 (1.14–3.24)	0.240
Liver	2.76 (2.20–3.27)	2.95 (2.56–3.44)	0.310
Spleen	0.89 (0.61–1.08)	0.81 (0.77–1.05)	0.818
Bladder	0.96 (0.38–2.74)	0.88 (0.64–1.22)	0.937

IC, intracoronary; RCVI, retrograde coronary venous infusion.

#### Ex vivo analysis

**Figure 4:**
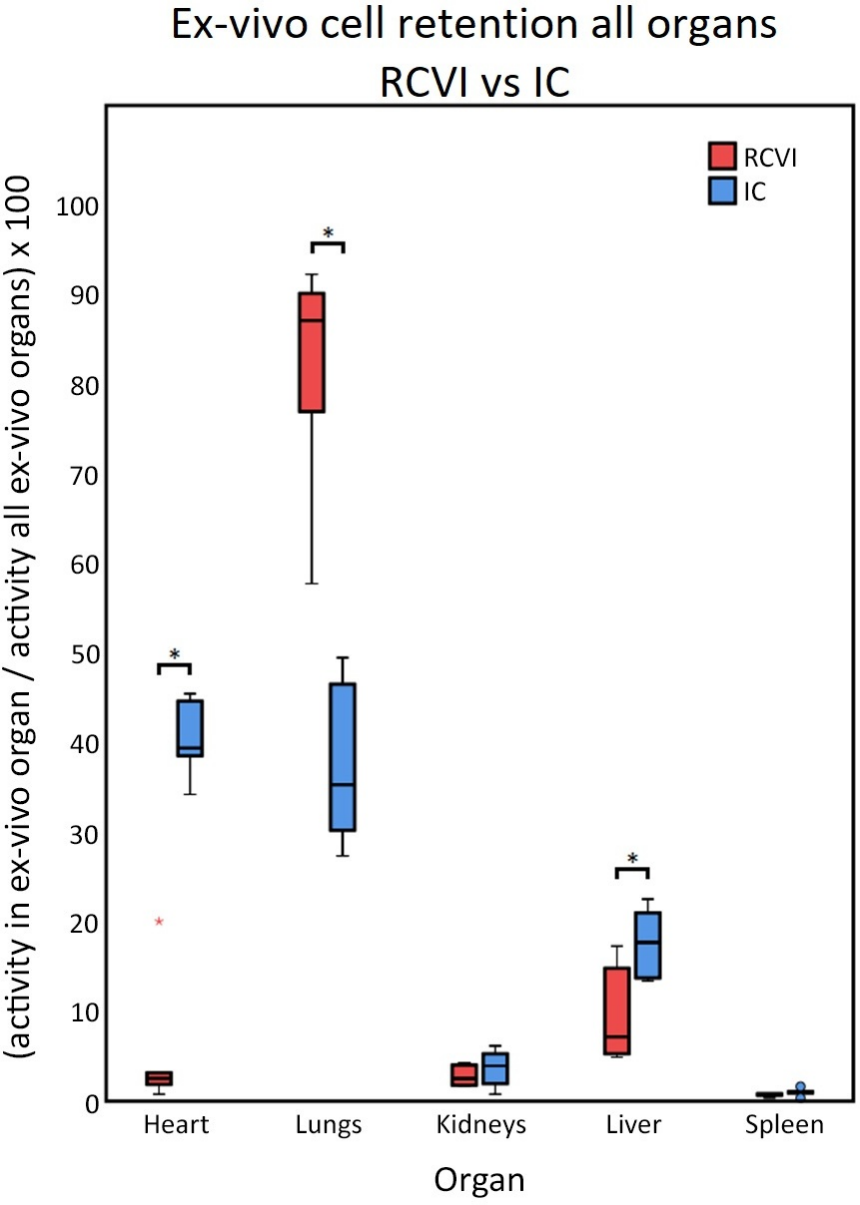
Ex vivo retention of cells in individual organs. Ex vivo analysis of activity in heart, lungs, kidneys, liver and spleen as a percentage of total activity from all individual organs combined. Activity in the heart, lungs and liver differed significantly between RCVI and IC infusion (p<0.05). IC, intracoronary; RCVI, retrograde coronary venous infusion.

In accordance with the in vivo results, a significant difference was seen in MSC retention in the heart between the RCVI and IC infusion groups after ex vivo analysis. The median retention was 2.55% (IQR: 1.86-3.16) in the RCVI group versus 39.40% (IQR: 38.54-44.64) in the IC group (p=0.002). Significant differences between RCVI and IC infusion were also seen for lung and liver retention (p=0.002 and p=0.04, respectively), with a significantly higher number of cells retained in the lungs after RCVI and a significantly higher number of cells retained in the liver after IC infusion. Data are represented in table 3 and figure 4.

**Table 3:** Ex vivo analysis of activity in heart, lungs, kidneys, liver and spleen as a percentage of the total counts coming from all individually scanned organs combined. Values are depicted as median with IQRs

Organ	Median activity (%) (IQR)	Median activity (%) (IQR)	P value
	RCVI (n=6)	IC (n=6)	
Heart	2.55 (1.86–3.16)	39.40 (38.54–44.64)	0.002
Lungs	87.10 (76.95–90.13)	35.32 (30.22–46.53)	0.002
Kidneys	2.53 (1.77–4.03)	3.95 (1.95–5.28)	0.394
Liver	7.17 (5.28–14.83)	17.71 (13.74–21.01)	0.041
Spleen	0.78 (0.64–0.84)	0.99 (0.89–1.08)	0.065

IC, intracoronary; RCVI, retrograde coronary venous infusion.

#### Histological analysis

**Figure 5:**
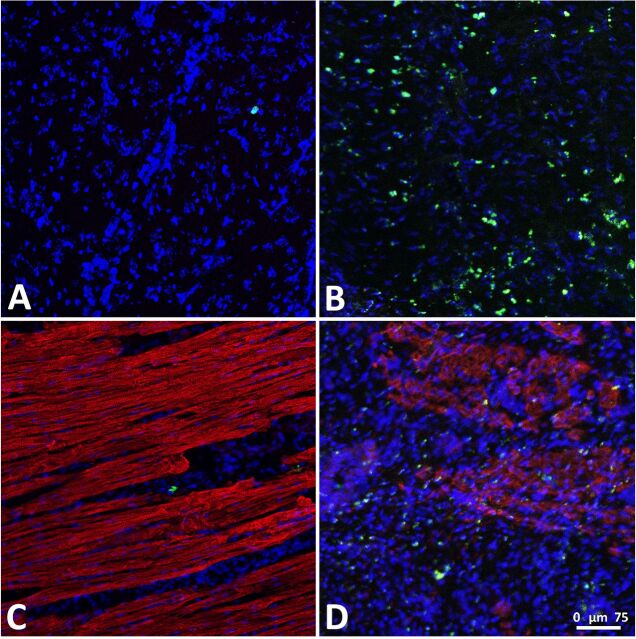
CFSE-positive cells in myocardial tissue samples. (A) Myocardial tissue sample after retrograde coronary venous infusion (RCVI), Hoechst staining and carboxyfluorescein succinimidyl ester (CFSE) signal. (B) Myocardial tissue sample after intracoronary (IC) infusion, Hoechst staining and CFSE signal. (C) Myocardial tissue sample after RCVI, Hoechst and α-actinin staining and CFSE signal. (D) Myocardial tissue sample after IC infusion, Hoechst and α-actinin staining, and CFSE signal. (Blue = Hoechst signal, Red = α-actinin signal, Green=CFSE signal).

Histology shows CFSE-labelled cells in the heart in the areas that are active on the scintigraphy. As expected, very few CFSE-positive cells were found in the myocardial tissue samples from pigs belonging to the RCVI group because myocardial cell retention was low in these animals. In line with our expectations, CFSE-positive cells were more abundant in tissue samples from the IC infusion group. Representative histological images are presented in figure 5.

### Safety aspects

#### RCVI group

Dissection of the CS occurred in three out of six pigs at the site of the balloon catheter tip. Two animals with the largest dissection later showed a radioactive hotspot in the heart instead of a more disseminated activity pattern as would be expected in case of cell infusion. Cardiac cell retention in these two pigs was the highest of all RCVI pigs and well above the median of 2.89% with 3.86% and 4.59% (in vivo data), respectively.

Three animals presented with a small-to-moderate, clear pericardial effusion and a haematoma of approximately 4 cm^2^ on the atrioventricular groove of the left ventricle (LV) at termination. Only one animal was free of dissection and development of haematoma and pericardial fluid. In this one animal, the occlusion of the CS was found to be compromised after the infusion was completed, possibly leading to direct drainage of cells into the right atrium. Nevertheless, the retention in this pig was 2.97% (in vivo data).

#### IC group

One animal in the IC group showed no flow directly after cell infusion, probably due to thrombus formation. Flow was restored after 5 min of angioplasty.

## Discussion

### Cell retention

The purpose of this study was to compare cardiac cell retention after RCVI and IC infusion and assess safety of RCVI. It was not possible to generate results on cardiac repair because the study design required termination of animals 4 hours after cell administration. To our knowledge, this is the first confirmatory study that directly compared retention between RCVI and IC infusion in a chronic MI pig model. We showed that RCVI of MSCs is inferior to IC infusion in terms of cardiac cell retention with RCVI showing a mean retention of 2.89% vs 13.74% with IC infusion (in vivo data). One can imagine that higher cell retention in the heart equals a greater effect of cell therapy, making IC infusion preferable over RCVI. We chose to infuse the same number of cells in both the IC infusion group and the RCVI group in order to make sure that the results are comparable. Because retention of cells in the heart is calculated as a percentage of the total administered cell dose in case of in vivo analysis, it is probable that a higher cell dose would not result in a higher retention rate. However, it is known that infusion of larger volumes of cells (30×10^6^–50×10^6^) via the IC route can result in a higher index of microcirculatory resistance.^17 18^

Currently, there is no evidence that larger cell volumes infused via the retrograde route would impair venous flow. This could implicate that more cells can be infused with RCVI, making up for the lower retention. The retention rates that we observed for IC infusion are comparable to results of other studies.^4 13 14^ Three pig studies with small sample sizes (n=5, n=6 and n=7) and only one clinical trial (n=9) reported retention rates after RCVI in a model of acute MI. However, no data on cell retention in a chronic ischaemia model in large animals are available. Retention of cells, measured as radioactive positive signals coming from the heart, was low in these four trials, ranging from 3% to 8% of total injected activity, corresponding with our results.^13 14 17 19^ A possible explanation for the low retention of cells with RCVI is that cells are maintained in the CS after infusion but are directly flushed into the right atrium after abrogation of the CS occlusion and reinstitution of flow. This could explain the higher retention of cells in the lungs of RCVI-treated animals. It is also possible that the cells are not adequately pushed though the microvascular bed as is the case with IC infusion, possibly effecting cell retention. Additionally, a low retention with RCVI could occur due to the existence of aberrant (and/or collateral) veins draining directly in the right atrium, effectively negating the blockade of the CS. An experienced cardiologist analysed the fluoroscopy images made during cell infusion in this study and found anatomical variations of the coronary veins strongly suggesting the presence of aberrant venous drainage in three out of six RCVI-treated animals. We could not find a relation between possible aberrant venous drainage and cell retention in these pigs, possibly due to the small number of pigs and other factors present such as CS dissection and pericardial effusion.

### Differences between in vivo and ex vivo data

With in vivo analysis we only found a significant difference in cell retention in the heart between RCVI and IC infusion, while we find a significant difference in cardiac, pulmonary and hepatic retention after ex vivo analysis. Also, a different magnitude of retention is seen between in vivo and ex vivo measurements.

To explain these differences, it is important to understand that cell retention in organs is calculated differently for the in vivo analysis and ex vivo analysis. In case of in vivo analysis, cell retention in a certain organ (numerator) is calculated as a fraction of total body activity (denominator). In case of ex vivo analysis, cell retention in a certain organ (numerator) is calculated as a fraction of total ex vivo organ activity (denominator). The difference in denominator between in vivo and ex vivo analysis means that in vivo and ex vivo data cannot be directly compared with each other. However, both analyses are relevant. In vivo data show the percentage of total administered cell dose that is retained in the heart. However, in vivo analysis has a few shortcomings. With in vivo analysis, ROIs are drawn around individual organs to determine the amount of radioactive counts coming from these organs. In this study, the ROIs were determined by experienced technicians and were accurately defined. However, there is always a margin of error with ROI definition for total body scans. When few cells are retained in an organ, the radioactive signal coming from this organ is low, making the contours of this organ difficult to discern from the surrounding tissue. This makes ROI definition more difficult in organs such as liver, spleen and kidneys, as seen in figure 2. Cardiac and pulmonary borders are usually easier to define, because of the higher retention in the lungs and heart. In case of low cardiac retention, as is the case after RCVI, the cardiac border is still easy to define because of the large difference of signal between the heart and surrounding lung tissue. A second drawback of in vivo total body imaging is overprojection of organs such as the heart and lungs. This could lead to overestimation of signal coming from the heart. Excision of organs followed by ex vivo scanning ensures that only counts coming from the individual organ are identified, overcoming errors caused by ROI definition and superposition of organs such as the heart and lungs. Thus, differences in significance of pulmonary and hepatic retentions between in vivo and ex vivo imaging can be explained by the difference in the way that retention is calculated, ROI definition and superposition of organs. However, total body scanning is the best option to determine the number of cells retained in the heart as a percentage of the total administered cell dose. With ex vivo analysis, the retention of cells in the heart cannot be expressed as a percentage of the total administered cells, because part of the activity and thus the cells are distributed outside of the organs, for instance in muscles and blood pool. For this reason, we decided to incorporate both in vivo and ex vivo data in this study. Both methods show that cell retention is significantly lower in the RCVI group compared with the IC infusion group.

### Safety aspects of RCVI

RCVI was associated with multiple safety issues in this study. We found pericardial fluid and haematoma development on the atrioventricular groove of the LV in three pigs and occurrence of CS dissection in three pigs, of which one also showed a haematoma and pericardial fluid at termination. Only one animal in the RCVI group was free of adverse events. It is striking that in this specific animal, the occlusion of the CS appeared to be incomplete after infusion. We do not know at which time point during the infusion procedure the occlusion was compromised.

In one animal, overinflation of balloon of the advance CS infusion catheter (>2 atmospheres) could have been the cause of development of a CS dissection, haematoma on the atrioventricular groove and pericardial fluid collection.

The most likely explanation for the development of pericardial fluid and haematoma is a sudden rise in pressure in the coronary venous system during infusion even though we infused cells slowly at 10 mL/min. Significant contrast blushing was seen on the fluoroscopy images made after infusion, supporting this hypothesis. We identified 10 studies that used RCVI for cell delivery in pigs.^14 17 19–26^ The median infused volume in these studies was 15 mL (IQR: 10–25 mL), with two studies infusing a higher volume of 40 mL^26^ and 250 mL.^19^ The study that infused 40 mL did so during 4 hours, making it likely that no pressure or volume overload could develop.^26^ However, the study that infused 250 mL did so during 10 min, making both the infused volume and infusion rate higher than in our study.^19^ Unfortunately, it is unclear if the CS was occluded during infusion in these two trials, so it is not possible to make a statement on pressure or volume overload in these cases. Three other trials infused cells at a much higher rate and did not report development of pericardial fluid and haematomas.^14 17 22^ However, the infused volume was only 10 mL in these three trials.

It is unfortunate that the majority of the RCVI pig studies reported did not state anything on procedural safety. The studies that do mention absence of arrhythmias and microvascular obstruction, but nothing on occurrence of dissection of the CS or development of haematomas or pericardial fluid. It is also possible that pericardial fluid collection and haematoma formation were related to CS injury in some of the cases. Contrary to RCVI studies, development of haematomas on the atrioventricular groove, pericardial fluid collections and damage to the CS have been reported in the field of cardiac surgery and have been related to traumatic catheter insertion, overinflation of the balloon in the CS and elevated CS infusion pressure during retrograde cardioplegia.^27–30^ With retrograde cardioplegia, the CS is accessed with a balloon-catheter to occlude the CS and subsequently infuse fluid to arrest the heart and protect the myocardium. This procedure is in a way comparable to RCVI. Injury to the CS, such as CS perforation or rupture, was reported to occur in 0.6% to 0.06% of the patients that underwent retrograde cardioplegia, essentially proving safety of this technique.^27 31^ A possible explanation for the high number of adverse events in the RCVI group in this study compared with an event rate of only 0.6% to 0.06% in human cases could be the difference in anatomy of the CS between humans and pigs. Contrary to humans, the hemiazygos vein drains in the CS in pigs. This leaves less room for balloon positioning in pigs, increasing the chance to perforate the CS with the catheter tip due to the small operating area. Clinical trials that have used RCVI did not report safety issues beside a rise in cardiac enzymes in some cases.^13 32–36^

The occurrence of CS dissections did not appear to have a negative effect on cell retention in the heart. On the contrary, the two pigs with the largest dissection showed the highest retention rates of all six RCVI pigs. It is likely that the infused cells collected between the wall layers of the dissected area, effectively trapping the cells and preventing them from washing out. IC infusion was associated with less safety issues with one animal showing no reflow directly after cell infusion, which could be restored within 5 min. Decreased blood flow after IC infusion is a known drawback and has been attributed to coronary embolisms leading to microvascular plugging in the past.^8–10^

### Future implications

Here, we found that retention rates with both RCVI and IC infusion are low (<14%), which may hamper the effectiveness of cell therapy. Therefore, alternative approaches to increase cell retention and survival are being investigated. These include the use of carrier materials such as nanomatrix gels, microspheres and cell sheets or patches,^37–39^ and pretreatment of grafted cells or target tissues, for instance by overexpressing prosurvival genes to increase survival of grafted cells in a hostile environment.^40 41^

## Conclusion

Cardiac cell retention after RCVI is significantly lower compared with IC infusion. Our results confirm previous research comparing retention of cells after RCVI with IC infusion in the setting of acute MI. Furthermore, RCVI presented with more safety issues than IC infusion. Taking both efficiency and safety into account, IC infusion is the preferred method of cell delivery between the two.
